# Big data, machine learning and artificial intelligence: a neurologist’s guide

**DOI:** 10.1136/practneurol-2020-002688

**Published:** 2020-09-29

**Authors:** Stephen D Auger, Benjamin M Jacobs, Ruth Dobson, Charles R Marshall, Alastair J Noyce

**Affiliations:** 1 Preventive Neurology Unit, Wolfson Institute of Preventive Medicine, Queen Mary University of London, UK; 2 Department of Neurology, Royal London Hospital, London, UK

**Keywords:** Neuroradiology, image analysis, health policy & practice, evidence-based neurology, clinical neurology

## Abstract

Modern clinical practice requires the integration and interpretation of ever-expanding volumes of clinical data. There is, therefore, an imperative to develop efficient ways to process and understand these large amounts of data. Neurologists work to understand the function of biological neural networks, but *artificial* neural networks and other forms of machine learning algorithm are likely to be increasingly encountered in clinical practice. As their use increases, clinicians will need to understand the basic principles and common types of algorithm. We aim to provide a coherent introduction to this jargon-heavy subject and equip neurologists with the tools to understand, critically appraise and apply insights from this burgeoning field.

## INTRODUCTION

The complexity of data used in clinical neurology is only likely to increase in the coming years as health records are digitalised and ‘data heavy’ technologies such as whole-genome sequencing become incorporated into routine clinical practice. Recent advances in artificial intelligence and the development of sophisticated machine learning algorithms offer a potential means to use these data more efficiently and effectively. However, a basic understanding of how these machine learning algorithms work is essential to help interpret and critically appraise their outputs, and so know what to believe.

## WHAT IS MACHINE LEARNING?

Machine learning algorithms process data in order to build mathematical models that in turn can be used to help make predictions and/or decisions. These algorithms are not explicitly programmed with instructions for how to solve a problem. Instead, they improve autonomously (or ‘learn’) from experience. The systems learn to generalise from example data, with minimal human intervention.

Machine learning is particularly useful when working with very large datasets (eg, EEG, MEG and most forms of imaging). It is well suited to tasks that require repetitive routine activity (such as interpreting scans) and, for some tasks, can perform faster and more accurately than a human interpreter.

## HOW MACHINE LEARNING ALGORITHMS WORK

There are many different types of machine learning algorithm, and the most appropriate algorithm depends upon the specific nature of the task at hand. Here, we briefly summarise three categories: supervised, unsupervised and reinforcement learning algorithms.

### Supervised learning

**Figure 1:**
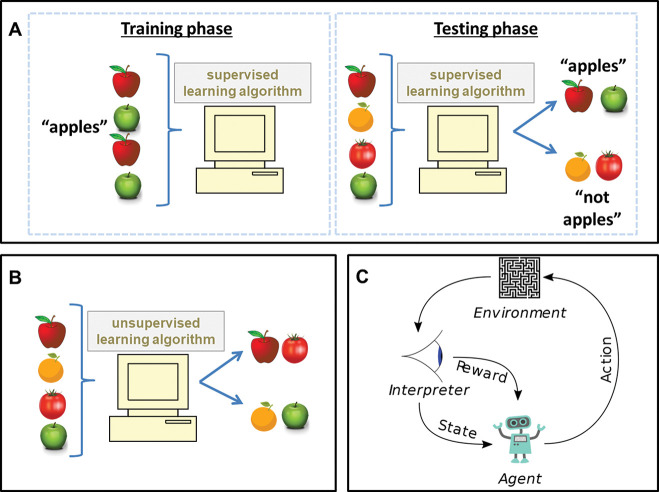
(A) Supervised learning algorithm. In the training phase, training data with associated labels are used to create a predictive model. In the testing phase, the predictive model is shown data that are not labelled, in this example to distinguish apples from other items. (B) Unsupervised learning algorithm. Unlabelled input data are processed by the algorithm to see what patterns it identifies, in this example to group red fruit. (C) Reinforcement learning algorithm. An agent performs an action in an environment. This is interpreted into a reward signal and a representation of the state, which are both fed back to the agent. Diagram in (C) reproduced from Wikimedia commons under public domain licence.

Supervised learning algorithms perform a complex ‘connect-the-dots’ operation between a given set of input data and an associated output. They are trained with multiple examples of a set of inputs and a paired known output, learning how to process the inputs in order to reproduce the related output. The fully trained algorithm can then be given novel sets of inputs, for which the outcome may not be known, and make a prediction as to what the related output should be (figure 1A). For example, an algorithm may be trained to classify whether the configuration of pixels in a picture (the input) represents an image of an apple (the output). Supervised algorithms are used for two types of problem: classification (to predict which ‘class’ an observation belongs to, eg, case vs control), and regression (to predict a continuous value, eg, time to diagnosis).

### Unsupervised learning

Unsupervised learning algorithms lack the ‘supervision’ of a known set of output information. They instead process input information to identify consistent patterns and associations between variables. The output they produce is a grouping or summary of the input data, rather than a targeted outcome (figure 1B).

### Reinforcement learning

Reinforcement learning algorithms are concerned with producing an *action* depending upon a given state. This ‘state’ refers to a configuration of inputs or observations from the environment the algorithm is operating in. They process a given state, decide upon an appropriate action based upon the state, observe the consequences of that action, and then ‘reinforce’ or penalise the state–action pairing accordingly (figure 1C). This makes the same action more or less likely for future scenarios in which a similar state is encountered. With multiple cycles of the same process, actions can be produced that maximise a rewarding outcome and minimise risk. The slow iterative nature of this process makes it well suited to tasks where there may be inconsistent or limited outcome events. For example, reinforcement learning algorithms have been used to great effect in optimising algorithms for playing chess and other board or computer games, where the ‘reward’ signal is fairly sparse or delayed that is, actions *eventually* lead to winning or losing the game, rather than receiving immediate feedback.

In summary, supervised learning algorithms are provided with a given set of results and have to sort data to reproduce them. Unsupervised learning algorithms are not provided with a set of results but still attempt to find patterns and associations within data. Reinforcement learning algorithms learn how best to react to a given state.

## ARTIFICIAL NEURAL NETWORKS

**Figure 2:**
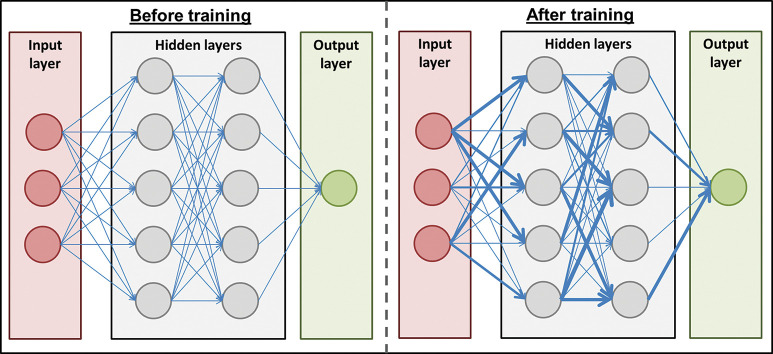
Artificial neural networks comprise neurons (circles) that are organised into an input (red), output (green) and any number of hidden (grey) layers. Neurons are connected with different weights of feed-forward connection between them (blue lines). Training the algorithm involves modifying the weights between neurons (represented above by the thickness of blue lines) so that connections associated with a rewarding outcome are strengthened and negative outcomes weakened.

Artificial neural networks refer to a structure of algorithm that loosely mimic their biological counterparts (figure 2). They are constructed of three types of layers of ‘neuron’: ‘input’ layer (analogous to primary sensory neurons), whose neurons ‘feed forward’ into any number of ‘hidden’ layers, which in turn feed forward to an ‘output’ layer. Neurons within a layer are not typically connected with one another but have multiple connections with neurons in the preceding or following layers. What a neurologist would understand as a synapse is termed a ‘weight’ between any two neurons. The structure of neurons and their layers are fixed, but the networks learn by adjusting these synaptic weights depending upon outcomes. A method called ‘backpropogation’ is a commonly used means for adjusting weights based upon how much error there was between the artificial neural network outcome layer’s prediction and the actual outcome (the ‘prediction error’). Whether the true outcome was unexpectedly rewarding or negative determines whether weights are either strengthened or weakened. The size of prediction error—that is, the mismatch between predicted output and ‘real’ output—determines the amount that weights are changed (ie, large prediction errors give rise to greater changes in weights).

Variations in the structure of an artificial neural network can allow for slightly different operations to be performed. Adding ‘self’ connections to neurons within a hidden layer (ie, a connection to and from the same neuron) give rise to ‘recurrent neural networks’, which can capture sequences of information to help in tasks such as speech or video analysis. ‘Convolutional neural networks’ are used to extract relevant features from an input. The process is somewhat analogous to, and was inspired by, the organisation of animal visual cortex (eg, edge detection in primary visual cortex). This helps filter and simplify complex inputs into the most salient features, which can allow for more efficient information processing.

So-called ‘deep learning’ algorithms are a form of artificial neural network that have many hidden layers, which allows them to perform more complex operations.

## LIMITATIONS OF MACHINE LEARNING ALGORITHMS IN CLINICAL PRACTICE

### ‘Junk in, junk out’

The performance of an algorithm depends heavily upon the quality of data available for it to learn from. Without a sufficient volume of high-quality training data, even the most state-of-the-art algorithms are doomed to failure. This is particularly relevant in clinical settings, where large volumes of high quality, labelled data are particularly difficult to come by.

This reliance upon large volumes of data was demonstrated by the reinforcement learning algorithm ‘AlphaGo’, which made the notable breakthrough of being the first machine able to defeat human players at the ancient board game ‘Go’. AlphaGo required training from a database of 30 million moves played by human experts followed by playing itself thousands of times over.^1^ There are a limited number of clinical questions amenable for providing datasets of n=30 million to help answer them. Subsequent refinements to AlphaGo relied purely upon simulated ‘self-play’,^2^ and again this sort of analysis of simulated outcomes to refine an algorithm’s performance would be less amenable to clinical applications.

### Bias and generalisability

**Figure 3:**
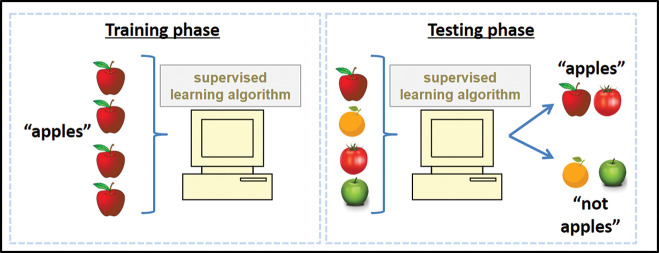
If a supervised learning algorithm has insufficiently diverse training data (eg, only apples which are red), then it is prone to misclassifying items that deviate from those narrow training data (eg, not identifying green apples or incorrectly labelling other red fruit, such as tomatoes, as apples). A more diverse training set, as in figure 1A, would be less prone to this sort of error.

An algorithm’s dependence upon the data available to it can also affect generalisability, and can help to perpetuate ingrained biases in health outcomes. For example, if an algorithm’s training dataset did not include a certain subcategory of a given disease, then there is no guarantee that it will be able to identify it correctly in future. This principle is illustrated with the example of apples in figure 3. An algorithm trained to detect the most subtle of subarachnoid haemorrhages on an MR scan might be completely blind to a large glioblastoma. Similarly, an algorithm trained to achieve high performance identifying pathology in images collected from a specific model of scanner may be blind to interpreting data from another. A model trained in an emergency department setting may not have the same performance characteristics in an outpatient clinic. Any deviation from the training conditions can lead to unpredictable behaviour from an algorithm.

If an algorithm is only ever exposed to cases of pathology in a specific patient demographic, it will be less likely to be able to identify similar pathology in other demographics. Most large cohorts, such as UK Biobank, will not fully represent the sampling population and indeed tend to be overrepresented by White and more socioeconomically advantaged groups.^3^ It is difficult to accrue truly diverse datasets to resolve this issue, giving a significant potential source of bias. Even if a diverse dataset is successfully acquired, systematic biases can also be reinforced by an overreliance upon an algorithmic approach. For example, a widely used algorithm in the USA that identifies people with complex health needs based upon healthcare costs, systematically underestimated illnesses of Black versus White people.^4^ This arose from pre-existing inequality in money spent caring for Black people compared with comparable White persons and led to a reinforced disparity in the help people of different races received.

**Figure 4:**
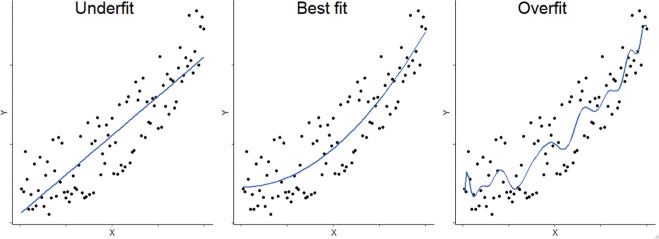
Schematic plots showing underfit (left) and overfit (right) models compared with the best fit (centre). The same data points are plotted (a quadratic function with random noise). The left panel shows a linear model, the right panel shows a high-order polynomial fitted to the data, the centre panel shows the quadratic function which was used to derive the data points. The model on the right panel is highly tuned to random noise in the data, and so is likely to perform poorly at predicting Y values from X values in an independent dataset. The linear model fits these data less ‘tightly’ (ie, there is a higher overall error), and so is less likely to predict Y values based on random noise but may be underfitted in that it does not capture some important structure in the data. The centre panel shows a quadratic function which captures the ‘true’ underlying distribution of the data and so is likely to perform best in an independent dataset.

To ensure generalisability, it is also crucial that an algorithm’s performance is validated on completely independent, ideally external, datasets. Unless validated upon independent data, it cannot be known whether an algorithm may be overfitting or underfitting. Overfitting refers to a scenario where the algorithm’s model fits too closely to the training data; that is, it learns the noise in the training data, to the detriment of its ability to estimate a relevant trend in an independent dataset. Underfitting is when the algorithm’s model does not capture the relevant trend in enough detail. For example, if the trend in data is best approximated by a non-linear curve (figure 4, centre panel), an underfitted model might estimate just a linear trend (figure 4, left panel), whereas an overfitted model might draw a line incorporating every individual datapoint rather than the smoothed best fitting curve (figure 4, right panel). If an algorithm’s performance drops significantly when tested on independent data, it suggests an element of underfitting or overfitting.

### Performance versus safety

Even if an algorithm can achieve commendable performance in classifying data, this does not necessarily equate to safety for its use in clinical practice. There needs to be consideration of *where* errors are made, not just how many are made on average. An algorithm that can classify 99% of stimuli correctly might on first appearances seem preferable to humans classifying with 96% accuracy. However, if the 1% incorrectly identified by an algorithm prove fatal and the 4% by humans benign, there are obvious implications for overall clinical safety. Clinicians are hopefully aware of rare but severe outcomes that are not to be missed; but with less exposure to rare but important outcomes, algorithms may be more liable to miss important results. The range of possible abnormal results is extremely broad and varied, making it difficult for training data to cover it comprehensively. Standard metrics of algorithm performance, such as accuracy, sensitivity and specificity, must be carefully interpreted in the clinical context and alone are not sufficient to ensure clinical safety. Clinical prediction algorithms that misclassify individuals could lead to dire consequences: healthy individuals misclassified as having a disease could be consigned to unnecessary and risky interventions; diseased individuals misclassified as healthy may be denied potentially helpful treatments.

It is important that algorithms are judged on clinical outcomes, in prospective trials rather than using simple performance metrics on retrospective data. At the time of writing, this has only so far been achieved in the context of colonoscopic polyp and adenoma detection.^5^

### ‘Black box’

While we know the input to an algorithm and the output it produces, there is often limited information about how they arrive at a particular solution; the so-called ‘black box’ problem of machine learning. This particularly relevant for deep-learning algorithms where there are a great many hidden layers that could be processing any number of features to pair an input and output. One notable example is an algorithm for classifying skin lesions as malignant or not, which picked up on the presence of rulers being situated alongside a lesion as more indicative of a malignant lesion.^6^ The ‘black box’ problem of machine learning algorithms means that significant confounding from any one of the many factors could easily be missed.

### Accountability

The introduction of algorithms into clinical practice is also fraught with potential ethico-legal implications. Who is to be held accountable if an algorithm makes an error with serious consequences—the person who wrote the code? the engineer who incorporated it into a device? the scientist who chose to apply it in a certain way? the regulator who approved it? the clinician who oversaw its use?

There are clear pathways for regulatory approval of novel drugs and devices, but algorithms pose potential new challenges. Not knowing why an algorithm may have made a particular decision (due to the ‘black box’ problem described above) complicates assessment of their safety and suitability for use in clinical practice. There is also the potential for machine learning algorithms to update, adapt and retrain dynamically in the face of new information. If an algorithm reaches a necessary threshold of performance to receive regulatory approval, updates from new information may be able to improve their safety even further, but the update would be a fundamentally new model. Would the pre-existing approval be able to apply to the new update? Or should this sort of rapid innovation be curtailed awaiting new regulatory approval? The pace of innovation in this field is likely to be significantly greater than rigorous regulation could ever be. Algorithms are currently regulated as medical devices, but dynamic software of this nature would struggle to comply with current change management processes. New models for the regulation of software are likely to be needed.

## APPLICATIONS IN NEUROLOGY

### Image classification

There is great commercial value for search engines, social media companies and automated vehicles to be able to extract meaningful semantic information from an image. Most of the early development of algorithms by commercial enterprises has focused on these sorts of task and image classification algorithms are at a more mature stage in their development than those used for most other tasks. This has had beneficial knock-on effects for algorithms looking to classify clinical images.

This has initially been explored in the triage of imaging to allow for rapid automated detection of potential abnormalities from large volumes of images. There are very large amounts of labelled imaging data available to help facilitate this (eg, CT scans of the head with linked radiology reports); it involves the sort of classification task that supervised learning algorithms do very well, and a role in triage minimises the overall responsibility placed upon an algorithm. Rapidly flagging potentially abnormal results to a human can provide genuine clinical benefit while maintaining a clinician as the ultimate decision maker. However, any benefit will need to be weighed with the potential for large numbers of false positives creating more work and distraction for already busy clinicians.

An area where a clear benefit of using machine learning algorithms is possible is the screening of optical coherence tomography imaging to determine appropriate onward referral. The widespread availability of this technique to opticians and in other healthcare settings has created a large volume of complex data that need analysing. This expansion of data has not been matched by a corresponding increased capacity of human expertise to interpret it. A supervised deep-learning algorithm has shown the potential to make referral recommendations matching those of experts for a range of sight-threatening retinal diseases.^7^ A volume of scans that would take humans days or weeks to get through could be analysed in minutes or hours by a machine learning algorithm (once it has been trained).

Analysis of retinal fundoscopic imaging has also been explored to assist triaging for whether referral for diabetic retinopathy might be necessary.^8^ Similar progress has also been made in analysing plain CT scans of the head^9 10^ to detect acute emergencies (eg, acute ischaemic stroke, subarachnoid haemorrhage, midline shift, mass effect or calvarial fractures).

Attempts have been made to use a semi-supervised learning algorithm to predict conversion of mild cognitive impairment to Alzheimer’s dementia.^11^ Using MR scans of people with mild cognitive impairment, the learning algorithm achieved 89% sensitivity and 52% specificity in predicting progression to Alzheimer’s disease based on images taken between 1 and 3 years before clinical diagnosis. Combining this imaging-based model with additional cognitive markers improved performance to sensitivity 87% and specificity 74%.

Recent work exploring the use of a supervised, deep-learning classification algorithm to detect papilloedema in ocular fundus photographs provides a good example of how careful and rigorous experimental design can minimise some of the limitations referred to above.^12^ The high performance of the algorithm for detecting papilloedema (96% sensitivity and 85% specificity) was as much a testament to the authors amassing an impressively large and diverse dataset, as to the sophistication of the algorithm itself. The dataset included 15 846 photographs from an ethnically diverse sample across 19 sites and 11 countries. Performance was not only validated upon independent data, but then also a completely separate external set of photographs obtained from different sites to those used for training the algorithm. Rather than assessing a simple papilloedema versus not papilloedema distinction, a third ‘other abnormalities’ condition limited the chance of the algorithm missing other clinically relevant findings that lay outside of the algorithm’s direct focus. As impressive as the performance of the algorithm was, to ensure its safety in routine clinical practice, ideally a prospective trial is needed to examine its use with images acquired in a non-specialist, non-trial setting (as would be its intended use).

### Electrophysiology interpretation

Interpretation of EEG recordings is another ‘data heavy’ area where machine learning has been employed. Extensive work has looked to develop algorithms to detect evidence of seizure activity.^13 14^ There is evidence that a combination of multiple deep-learning algorithms could be used to analyse EEG traces and identify personalised signatures of a pre-ictal brain state up to an hour before a seizure.^15^ This could have important implications for identifying and mitigating a seizure before it produces symptoms.

Machine learning has been used in the diagnosis and prognosis of coma. Supervised learning algorithms working on EEG recordings have been used to discover evidence of covert consciousness in unresponsive individuals following an acute brain injury.^16^ Among individuals who were not responsive to spoken commands, up to 15% had detectable brain activity in response to these commands. A deep-learning artificial neural network analysing EEG data in comatose patients 12 h following cardiac arrest was able to predict 6-month functional outcome: good (48% accuracy and 0% false-positive rate) versus bad (58% accuracy and 5% false-positive rate).^17^

Work in this area has also begun to go beyond passive recording and classification of inputs with the development of brain–machine interfaces that offer the intriguing potential to one day to communicate with people in a coma.^18^

### Treatment decisions

Other areas of neurology rely on the interpretation of much more messy and imperfect data. In the near future, machine learning realistically will be available only as a tool to assist in diagnosis rather than to make treatment decisions independently. However, work in other fields has considered using reinforcement algorithms to assist in areas such as heparin dosing^19^ and radiotherapy planning.^20^

### Future areas

Machine learning approaches will not replace neurologists but they may become an important aid in the diagnosis, prognosis and management of neurological disorders. Machine learning could enhance clinical practice in several areas (box 1):

**Box 1**

- Diagnosis.
  - Video classification:
    - Seizure classification: for example, epileptic vs non-epileptic seizures, phenotype in complex genetic epilepsies.
    - Tremor classification.
  - Image classification.
    - Syndromic diagnosis (for rare genetic disorders).
    - Neuro-cutaneous syndrome diagnosis.
    - Pathological diagnosis (biopsy or post-mortem).
- Genetic applications.
  - Prediction of novel disease-causing variants in rare genetic disorders (eg, from whole-genome or whole-exome sequencing).
  - Enhancing prediction of complex polygenic disease (eg, Parkinson’s disease, migraine, multiple sclerosis) by modelling non-linear interactions between many thousands of genetic variants in polygenic risk scores.
- Epidemiology.
  - Detecting ‘signatures’ of prodromal/early disease in large population-based datasets, for example, Clinical Practice Research Datalink (CPRD), UK Biobank.
- Management.
  - Predicting response to therapeutics.

The quality of available datasets is paramount in order to take full advantage of advances in machine learning technology. To benefit from the technology properly, neurologists will have to create datasets that provide a consistent standardised coding of relevant information from as diverse a range of clinical settings, patient characteristics, disease phenotypes and outcome measures as possible.

To date, development of machine learning algorithms has mainly focused on training them to reproduce diagnoses that are made by humans or panels of human experts. If they are to achieve a performance surpassing that of humans then the data used to train algorithms would have to change. If the ‘ground truth’ output used to train models consists of diagnoses made by humans, there is a ceiling to the maximal performance that a machine could achieve. Supra-human performance would be only achievable if the inputs to an algorithm are paired to an output that humans cannot predict; for example, matching CT images or a clinical phenotype to subsequent biopsy results or long-term outcome data. This clearer matching of routine investigations and disease phenotypes with important outcome data might also assist human decision makers, not just their artificial counterparts.

In an unsupervised learning setting, machine learning algorithms may be able to categorise distinct disease phenotypes better than humans: this may become especially relevant in the diagnosis of neurodegenerative disorders, in which there is often a poor correlation between clinical features and post-mortem pathological diagnosis. Just as biological neural systems have informed the development of their artificial counterparts, artificial networks are now being used to try and inform our understanding of natural processes.^21 22^

**Key points**

- Machine learning algorithms can be classified broadly into those performing supervised, unsupervised and reinforcement learning.
- The recent development of ‘deep learning’ algorithms allows for complex operations on very large datasets.
- There are many limitations that need careful consideration when trying to apply machine learning approaches in clinical practice.
- Image classification and triage are suitable initial applications for machine learning approaches.

**Box 2 Glossary**

- **Machine learning**: the use of algorithms (models) to make predictions or detect patterns in data, where the algorithm or model uses features of the data to enhance performance.
- **Training set**: the data used to refine the parameters of the model.
- **Test set**: also called ‘validation set’. Ideally, a completely independent dataset used to determine whether the trained model can perform well on new data it has not ‘seen’ before.
- **Overfitting**: a problem that arises when a model is too highly attuned to granular variation in the training set. Although it performs well in the training set, it performs poorly on new data as its predictions are too heavily influenced by noise in the training set, rather than the ‘ground truth’ structure of the data.
- **Supervised learning**: the training set includes ‘ground truth’ labels, that is, the outcome of interest is known and can be used to quantify the accuracy of the predictions.
- **Unsupervised learning**: rather than predicting labelled outcomes, the algorithm is trained to detect patterns or structures in the data.
- **Observation**: one ‘row’ of data, usually referring to an individual person or reading.
- **Variable**: one ‘column’ of data. A property of an individual observation.
- **Feature**: a property of data that can be used to make predictions. Features can be individual variables, transformed variables, derived variables, or complex composites of individual variables. ‘Feature extraction’ and ‘feature processing’ refer to steps applied to the data prior to training of the model. Aims of feature extraction/pre-processing are to maximise the informativeness of the available data, minimise random noise, and maximise computational efficiency (high-dimensional data can be reduced to more compact representations, using methods such as principal component analysis).

## FURTHER READING/MEDIA

1 Milea D, Najjar RP, Jiang Z, *et al.* Artificial intelligence to detect papilledema from ocular fundus photographs. *N Engl J Med* 2020;382(18):1687–1695.
  - an example of a rigourously designed trial which uses a machine-learning algorithm in a clinical setting, accounting for many of the technology’s limitations.
2 De Fauw J, Ledsam JR, Romera-Paredes B, *et al.* Clinically applicable deep learning for diagnosis and referral in retinal disease. *Nat Med* 2018;24(9):1342–1350.
  - a study which demonstrates the powerful potential of machine learning to create important efficiencies in clinical data processing.
3 AlphaGo—The Movie—https://www.youtube.com/watch?v=WXuK6gekU1Y
  - A documentary which demonstrates the significance of recent advances in machine learning technology outside of a clinical setting.
